# RETRACTION: Effect of Fast‐Track Surgery on Postoperative Wound Pain in Patients With Prostate Cancer: A Meta‐Analysis

**DOI:** 10.1111/iwj.70354

**Published:** 2025-03-18

## Abstract

**RETRACTION:** FanS. , LiuH. , ZhuY. , ZhengZ. , and CuiQ. , “Effect of Fast‐Track Surgery on Postoperative Wound Pain in Patients With Prostate Cancer: A Meta‐Analysis,” International Wound Journal 21, no. 2 (2024): e14417, 10.1111/iwj.14417.PMC1082469937737032

The above article, published online on 22 September 2023, in Wiley Online Library (http://onlinelibrary.wiley.com/), has been retracted by agreement between the journal Editor in Chief, Professor Keith Harding; and John Wiley & Sons Ltd. Following an investigation by the publisher, all parties have concluded that this article was accepted solely on the basis of a compromised peer review process. The editors have therefore decided to retract the article. The authors did not respond to our notice regarding the retraction.

**RETRACTION:** S. Fan , H. Liu , Y. Zhu , Z. Zheng , and Q. Cui , “Effect of Fast‐Track Surgery on Postoperative Wound Pain in Patients With Prostate Cancer: A Meta‐Analysis,” International Wound Journal 21, no. 2 (2024): e14417, 10.1111/iwj.14417.PMC1082469937737032

The above article, published online on 22 September 2023, in Wiley Online Library (http://onlinelibrary.wiley.com/), has been retracted by agreement between the journal Editor in Chief, Professor Keith Harding; and John Wiley & Sons Ltd. Following an investigation by the publisher, all parties have concluded that this article was accepted solely on the basis of a compromised peer review process. The editors have therefore decided to retract the article. The authors did not respond to our notice regarding the retraction.

